# Commercial Brain Training: Efficacy, Transfer Effects, and the Influence of Personality Traits: A Study Conducted on Healthy Young Adults

**DOI:** 10.3390/brainsci11081083

**Published:** 2021-08-18

**Authors:** Florian Scholl, Sören Enge, Matti Gärtner

**Affiliations:** 1 Department of Psychology, Faculty of Natural Sciences, Medical School Berlin, 14197 Berlin, Germany; soeren.enge@medicalschool-berlin.de(S.E.); matti.gaertner@medicalschool-berlin.de (M.G.); 2 Department of Psychiatry and Psychotherapy, Campus Benjamin Franklin, Charité—Universitätsmedizin Berlin, 12203 Berlin, Germany

**Keywords:** cognitive training, working memory, personality, transfer effects, episodic memory

## Abstract

In the present study, we investigated the effects of a four-week working memory (WM) and attention training program using commercial brain training (Synaptikon GmbH, Berlin). Sixty young healthy adults were assigned to the experimental and active control training programs. The training was conducted in a naturalistic home-based setting, while the pre- and post-examinations were conducted in a controlled laboratory setting. Transfer effects to an untrained WM task and to an untrained episodic memory task were examined. Furthermore, possible influences of personality, i.e., the five-factor model (FFM) traits and need for cognition (NFC), on training outcomes were examined. Additionally, the direct relationship between improvement in single trained tasks and improvement in the transfer tasks was investigated. Our results showed that both training groups significantly increased performance in the WM task, but only the WM training group increased their performance in the episodic memory transfer task. One of the training tasks, a visuospatial WM task, was particularly associated with improvement in the episodic memory task. Neuroticism and conscientiousness showed differential effects on the improvement in training and transfer tasks. It needs to be further examined whether these effects represent training effects or, for example, retest/practice or motivation effects.

## 1. Introduction

The digitization of our daily lives is characterized by ever increasing cognitive demands. This is accompanied by a growing interest in pharmacological and non-pharmacological cognitive enhancement strategies [1,2]. Brain training games are a supposedly attractive way to improve cognitive performance because no side effects are assumed, and they are nowadays easily accessible via numerous mobile applications. Furthermore, brain imaging studies suggest that cognitive brain training promotes neural plasticity and leads to increased activation in task-relevant brain regions [3]. However, are (commercial) brain training applications really effective, and crucially, is there a transfer from trained to untrained tasks? While training developers promise a significant increase in cognitive abilities, the empirical evidence of the effectiveness of cognitive brain training is still pending [4].

An early study that received much attention suggested that fluid intelligence (Gf) could be improved through working memory (WM) training [5]. However, subsequent studies failed to reproduce this effect [4], and according to the current state of research, it is unlikely that this type of far transfer, i.e., transfer from one cognitive domain to another, can be achieved through cognitive brain training [6,7,8,9]. Nevertheless, the study by Jaeggi et al. [5] promoted interest in the field and several studies have investigated different forms of training and transfer effects since then. Across studies, it appears that the possibility of so-called near transfer, i.e., transfer to untrained tasks, which are similar to the trained one, may exist [10]. However, there are only moderate effects for near transfer and almost none for far transfer [11]. For example, near transfer from a trained WM task to a verbal complex span task [10] to structurally similar WM tasks [12] and to WM updating [13] have been reported. In addition, near transfer effects of a WM span training to free recall memory tasks have been observed [14].

Although a recent meta-analysis criticizes that studies with the most striking results often do not include active control groups, near transfer effects are considered likely to occur [8]. Proceeding from this, it seems necessary to further investigate to what extent transfer from trained to untrained tasks is possible, and whether brain training enhances cognitive performance in everyday life.

The results of one study suggest that WM training increases performance in an episodic memory task [15]. Effects of an improvement of episodic memory in the elderly through a commercially available brain training program could also be observed [16]. This type of transfer could have important positive implications, such as on general well-being [17].

Previous research on cognitive brain training has often been conducted under laboratory conditions, which, however, may limit external validity. It is therefore reasonable to examine commercial brain training programs, which have a wide range and applicability in everyday life, making brain training accessible to the public, for their effectiveness of WM training. Strobach and Huestegge [18] applied a commercial brain training application named NeuroNation.com (accessed on 1 June 2020) provided by Synaptikon GmbH, Berlin [19] in a naturalistic setting and found a significant overall performance increase in the trained tasks. In addition, they found significant differences between the experimental and control groups in the trained tasks and also observed significant near transfer effects. Meta-analytic findings on the commercial training program Cogmed join the general state of research on working memory training. Almost no far transfer effects could be found across all studies. However, small to medium effects were found in the area of near transfer [20]. In a broad cross-sectional study across different cognitive brain training programs, training effects were also found for working memory tasks [21]. There have been fewer studies to date in settings that are as naturalistic as possible, but particularly noteworthy here is the study by Stojanoski, Wild, Battista, Nichols, and Owen [22].

In previous research, *n*-back tests have been frequently used as WM training tasks. In *n*-back tasks, a continuous stream of items is presented to participants who must decide whether a given stimulus matches an item that was presented *n* trials before [23]. Exercises based on *n*-back tasks can, for instance, also be found in commercial brain training [18].

Given the mixed results of studies on WM training and associated transfer effects, the question arises whether these results could also be due to the influence of personality traits. Interestingly, Studer-Luethi, Jaeggi, Buschkuehl, and Perrig [24] found that participants with higher levels of neuroticism showed less training efficacy and thus performed worse in a dual *n*-back task than those with lower levels. In the same study, conscientiousness was associated with larger training gains and improved near transfer in a single *n*-back task. Further findings on neuroticism showed that higher neuroticism led to lower performance in complex WM tasks [25].

This is consistent with conceptual views that neuroticism increases responses to stressors and the probability of how often and intensely emotions of fear, anger, depression, frustration, etc. are felt by an individual. Conscientiousness, in turn, describes the need to manage a given task as well and correctly as possible and to fulfill one’s obligations [26]. The other three traits of the five-factor model (FFM), i.e., openness, extraversion, and agreeableness, are exploratively examined in the present study. Moreover, the need for cognition (NFC) was investigated, which is defined as the intrinsic motivation to engage in and enjoy effortful cognitive endeavors [27] and may thus influence how engaged participants are in the training tasks.

In the present study, we applied the commercially available WM training program NeuroNation.com (accessed on 1 June 2020) in a naturalistic training setting and investigated transfer effects to an untrained WM task and to an untrained episodic memory task. Training and transfer effects were compared to the results of an active control group that performed an attention training program using the same commercial training application. Both training programs were adaptive and included several different training tasks. The tasks in the WM training program focused on different aspects of WM such as short-term memory, updating, memory span, and spatial WM. The first transfer task was a visual *n*-back task, which is a continuous working memory task with aspects of maintaining and updating information in working memory. These aspects of WM were trained in the training tasks, but the combination of continuous updating and maintaining of information as it is implemented in the *n*-back task was not trained. As such, the visual *n*-back task meets the criteria of a near transfer task. The second transfer task (episodic memory) was not designed as a pure working memory task. Between the encoding and retrieval phase, a distractor task was implemented to actively prevent storage in short-term memory. Additionally, participants were encouraged to make use of elaborative mnemonic strategies such as a method of loci. This encoding aspect was not directly present in the training tasks. However, some of the described aspects of the training tasks, such as spatial working memory (e.g., PathMemo), or associative memory (e.g., Symbolski), might have been indirectly beneficial to successfully perform the word list task. To the best of our knowledge, this type of transfer is not yet clearly defined in the literature. On the one hand, it is not a near transfer since untrained encoding strategies are needed to perform the transfer task. On the other hand, the transfer task is in the same cognitive domain (i.e., memory), so it is not far transfer either. The tasks trained in the active control group focused on different aspects of attention such as sustained and selective attention, as well as processing speed. However, it should be noted that due to the choice to use commercially available training programs, there is a certain overlap between the two training programs. Nevertheless, there is clear WM focus and attention focus in the two respective training programs. A full description of the training and transfer tasks is provided in the Section 2 and the Supplementary Materials.

Exploratively, we investigated which of the training tasks was directly linked to performance increases in the transfer tasks after training. Additionally, we examined whether and how certain personality traits may influence training and transfer effects in the present study.

## 2. Materials and Methods

The participants were recruited in 2019 and 2020 at Medical School Berlin (MSB). The sample consisted of healthy young university students who received course credit for their participation. A total of *n* = 60 participants aged 19 to 37 years were involved in the study. Forty-four of these participants defined their gender as female and 16 of these participants as male. The average age was 22.22 (*SD* = 2.53) years. This study was conducted in accordance with the Declaration of Helsinki (revised form). All participants were informed in advance about the course of the study, the voluntary nature of the study, and data protection and anonymity and signed a written declaration of consent. The participants received course credit for participating in the experiment if they completed at least 14 of the 28 training units.

The German version of the Big Five Inventory 2 (BFI-2) was used [28] to assess the five-factor personality traits according to Danner et al. (2016), and the internal consistency (Cronbach’s alpha) for extraversion is *α* = 0.85, for agreeableness *α* = 0.80, for conscientiousness *α* = 0.86, for neuroticism *α* = 0.89, and for openness *α* = 0.85. The NFC was assessed using the German version [29]. According to Bless, Wänke, Bohner, Fellhauer, and Schwarz et al. [28], the internal consistency (Cronbach’s alpha) is *α* = 0.86. The near transfer effect was measured by a visual *n*-back task as reported by Gärtner, Rohde-Liebenau, Grimm, and Bajbouj [30] in their study.

A 3-back task was used where a response by pressing the spacebar was required if a certain number presented in the center of a computer screen matched a number presented three stimuli before. There were several trials with a ten second break in between. Each trial consisted of a sequence of 30 consecutive digits (1, 2, 3, and 4). Digits were shown for 400 ms each, followed by a pause of 1500 ms, until the next digit appeared. A total of 300 stimuli were presented, of which 100 were targets and 200 were non-targets. The numbers were presented in a pseudo-random sequence with a separate version used for both measurement time points. There was no feedback on the accuracy.

To measure the transfer effect to episodic memory, a word list task as reported in Gärtner and Bajbouj [31] was used. Here, 20 words were visually presented in six trials and had to be remembered correctly after a distraction task. The distraction task was designed to prevent words from being retained in the phonological loop [32] and consisted of four calculation tasks taking approximately one minute. To avoid training effects between baseline measurement (t0) and follow-up measurement (t1), there was a counterbalancing of the word lists. For this purpose, six comparable word lists for pre- and post-assessment were created. The 20 nouns were presented at a rate of 2.75 s. The nouns appeared on the computer screen for 1000 ms, followed by a fixation cross in the center of the screen presented for 1750 ms. Following the distraction task, there was another minute of free recalling the nouns from the previously presented word list. All nouns were taken from the “Berlin Affective Word List” [33]. Again, there was no direct feedback on whether the words were remembered correctly.

The NeuroNation.com training program from Synaptikon GmbH, based in Berlin, was used for cognitive brain training. Two four-week training programs, as provided by NeuroNation.com, were selected. Both programs consisted of ten different task types [19]. The WM training program focused on classical WM tasks and the attention training program focused on processing speed and attention. The training was performed using the desktop version. Each unit consisted of five domain-specific tasks and the processing took place online and privately. Both programs were designed as adaptive training, in which the difficulty increases constantly with successful completion of the tasks and decreases vice versa. A representative task for the WM program is the *PathMemo* task (see Supplementary Materials). This task design focuses on visuospatial memory and WM span. Moving circles must be tapped in the previously displayed order. A representative task for the attention program is the *Mackworth* task (see Supplementary Materials). A dot moving in a clock-like circle must be tracked, and whenever it skips a field, a response is to be given. Due to the predetermined training programs, individual working memory tasks can also be found in the attention program and vice versa, in fact, three tasks have been part of both training programs.

Prior to the experiment, information about the experiment was provided and written informed consent of the participants was obtained. The experiment used an experimental and an active control group. The participants were randomly assigned to the groups (blind to the investigators). Data were obtained at two points in time, t0 and t1.

The experimental group was assigned to the WM training program. The active control group was assigned to the attention training program. During baseline measurement, all participants filled in the BFI-2. Potential transfer effects on WM were measured by the *n*-back (near transfer) test and episodic memory by the word list task. The pre- and post-measurements were conducted in the laboratory at the MSB. Both groups performed the training for about 15 min, using the desktop version at home, daily, over a period of four weeks. During the subsequent follow-up measurement, the *n*-back task and the word list task were completed.

Data analyses were performed using SPSS (Version 25, IBM, Armonk, NY, USA).

Participants who had completed at least 5 training sessions and had no substantial prior experience with cognitive brain training were considered in the analysis. Of the participants, 83.3% reported no prior experience with cognitive training at all at the beginning of the study. The remaining 16.7% reported on average 3.89 (*SD* = 3.66) hours of prior training experience. It was assumed that a few completed training sessions would not lead to a noticeable improvement.

To investigate transfer effects, a mixed model ANCOVA with time (pre, post) as the within subject factor and group (memory, attention training) as the between subjects factor was implemented. The dependent variable was the performance in either the *n*-back task or the episodic memory task. Age and performance at baseline were included as covariates. This approach effectively controls for potential baseline performance differences between participants. The *n*-back performance was calculated as accuracy in percent, i.e., number of hits, minus the false alarms, divided by the targets, multiplied by 100. The mean reaction time (RT) of the task was measured in milliseconds. The performance in the episodic memory task was calculated as the number of correctly remembered words in the word list task.

The improvement in the training tasks was calculated as the following: the algorithm of NeuroNation.com ensures adaptive training and dynamically changes the task types depending on the performance of the participant and the session number. Therefore, the tasks were performed in different frequencies across participants. In order to obtain useful comparable data for the analysis, only tasks that were performed more than six times by more than ten participants were considered. The mean score of the first two runs of each task was defined as the baseline value for analysis, and the last run was used to calculate the improvement during training. The mean score across all tasks was calculated as average improvement. Detailed figures and statistics for the individual training tasks are provided in the Supplementary Materials. Due to corrupted logfiles, training data were missing for two participants and training data were available for 28 participants in the WM training group and 24 participants in the attention training group. The personality traits were operationalized as a mean score from the BFI dimensions neuroticism, conscientiousness, openness, extraversion, and agreeableness. NFC was operationalized as a mean score as well.

Correlational analyses (Pearson) of personality variables with the average improvement of the WM training and attention training tasks as well as with the improvement of the *n*-back and word list tasks were performed. The improvement in the *n*-back task was calculated as the difference in *n*-back accuracy between t0 and t1. The improvement in the word list task was calculated as the difference in correctly remembered words between t0 and t1.

## 3. Results

The final sample consisted of *n* = 54 participants who completed baseline and follow-up measurement and the minimum amount of required training sessions, as outlined above. Forty-two of them defined themselves as female (77.8%), while 12 defined themselves as male (22.2%). The experimental group consisted of 29 participants with an average age of 21.83 years (*SD* = 1.69). The active control group consisted of 25 participants with an average age of 22.12 years (*SD* = 1.74). A total of 17.31 (*SD* = 6.26) training units were completed on average. The experimental group completed an average of 17.34 (*SD* = 6.58) training units. Analyses of the training data showed that in the WM group, eight different tasks had been performed at a sufficient frequency. On average, analyzed tasks were performed by *M* = 23.38 (*SD* = 2.88) participants, and on average the tasks were performed *M* = 9.99 (*SD* = 2.58) times. Performance increased substantially in and across all tasks (all *p* < 0.001). In the attention group, eight different tasks were performed at a sufficient frequency. On average analyzed tasks were performed by *M* = 21.25 (*SD* = 2.12) participants, and on average the tasks were performed *M* = 9.99 (*SD* = 3.00) times. Performance increased in all tasks (all *p* < 0.001). The control group completed an average of 17.28 (*SD* = 5.99) training units. There were no significant group differences for any of the variables analyzed (all *p* > 0.05; see Table 1).

**Figure 1:**
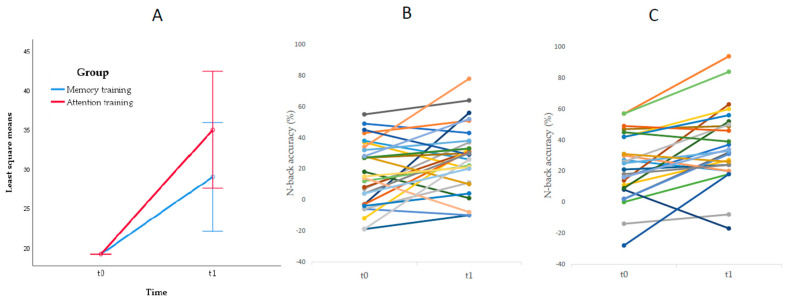
Improvement in *n*-back task. (**A**) Mean improvement in *n*-back accuracy by training program, (**B**) improvement in *n*-back accuracy by participant and measurement point for working memory program, (**C**) improvement in *n*-back accuracy by participant and measurement point for concentration program, *N*(t0, t1) = 54.

The ANCOVA conducted to test near transfer effects showed a significant effect of time, *F*(1, 50) = 4.95, *p* = 0.031, *ηp*2 = 0.090, and no significant interaction effect between time and group, *F*(1, 50) = 1.38 *p* = 0.246, *ηp*2= 0.027 (see Figure 1).

**Figure 2:**
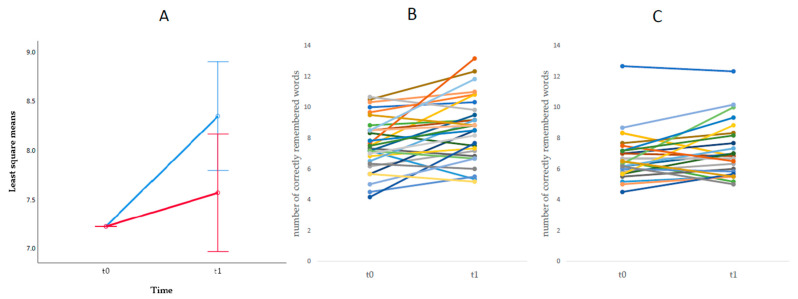
Improvement in word list task. (**A**) Mean improvement of correctly remembered words by training program, (**B**) improvement in correctly remembered words by participant and measurement point for working memory program, (**C**) improvement in correctly remembered words by participant and measurement point for concentration program, *N*(t0, t1) = 54.

The ANCOVA for the episodic memory performance showed no significant effect of time, *F*(1,50) = 1.35, *p* = 0.25, *ηp*2= 0.026, but a significant interaction effect of time and group, *F*(1, 50) = 4.60, *p* = 0.037, *ηp*2= 0.084 (see Figure 2). Post hoc *t*-tests showed a substantial performance improvement in the WM group, *t*(28) = −3.43, *p* = 0.002, while no significant improvement was observed in the attention group, *t*(24) = −1.64, *p* = 0.12.

**Figure 3:**
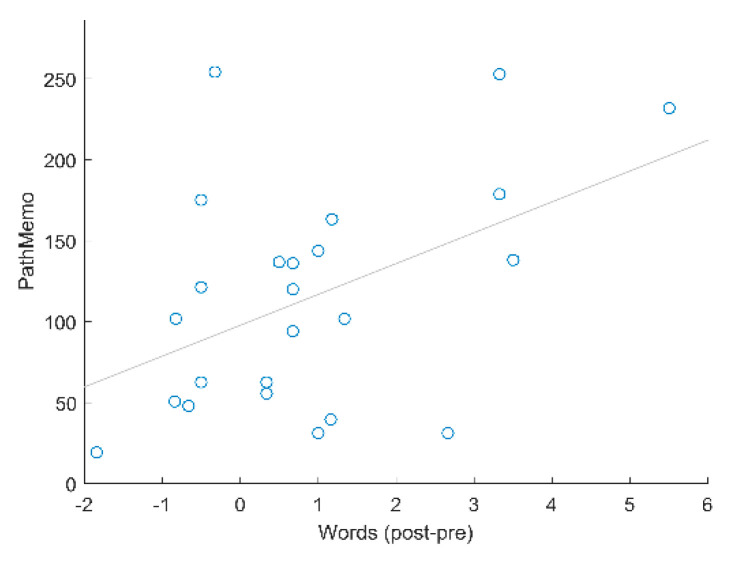
PathMemo performance and improvement in episodic memory transfer task.

In a merely exploratory analysis, performance increases in the individual tasks were correlated (Pearson’s correlation coefficient) with performance increases in the transfer tasks. Interestingly, the only significant relationship was observed in the WM group, between improvement in the PathMemo task and improvement in episodic memory performance, *r*(22) = 0.47, *p* = 0.021 (all other *p* > 0.1) (see Figure 3). For all of the analyses, no alpha error corrections were performed.

The analysis of the effects of personality traits revealed that neuroticism had a significant positive effect in the WM training tasks, *r* = 0.39, *p* = 0.048, and a negative trend effect, *r* = −0.35, *p* = 0.060, on the improvement in the *n*-back transfer task, while there was no significant effect, *r* = −0.01, *p* = 0.974, in the word list task. In the attention training, serving as active control group, a similar positive relationship of *r* = 0.29, *p* = 0.195 was observed as well as a negative one for the *n*-back transfer task, *r* = −0.30, *p* = 0.144, and the word list transfer task, *r*= −0.44, *p* = 0.029.

Regarding conscientiousness, no substantial effects on the WM training tasks, *r* = −0.06, *p* =0.756, but a significant effect on the improvement in the *n*-back transfer task, *r* = 0.43, *p* = 0.020, were observed. Moreover, a positive association was found for the untrained word list transfer task, *r* = 0.30, *p* = 0.115. In the active control group, conscientiousness showed neither effects with statistical significance nor trend effects or those of substantial effect size on the training and transfer tasks (all *p* > 0.20).

The traits agreeableness and openness as well as the NFC showed no systematic effects in the WM training and transfer tasks, while extraversion showed a significant negative association solely for the WM training tasks *r* = −0.41, *p* = 0.039.

## 4. Discussion

The present study focused on transfer effects after a four-week training program with a commercially available brain training application in a naturalistic training setting. The experimental group received an adaptive WM training program and an adaptive attention training program served as an active control group. In addition to replicating near transfer effects on an untrained Figure S1d WM task as reported by previous studies, we focused on potential transfer effects to episodic memory. Furthermore, we investigated the possible moderating role of fundamental personality factors on training and, in an exploratory manner, which of the trained tasks were directly associated with observed transfer effects.

Our results showed that participants in both training programs substantially increased the performance in the trained tasks. Furthermore, a significant performance increase for the near transfer task in both training programs, but with no differences between groups, was observed. One possible explanation for the improvement in both groups could be that both groups actually benefited from the respective trainings. Attentional resources are as important as WM capacity for successful performance of the *n*-back task [34]. However, due to the lack of a passive control group, retest effects cannot be excluded. The improvements found in the *n*-back task may be seen in the context of the previous literature. Salminen et al. [13] reported training-induced WM improvements in the visual and auditory *n*-back task. Linares et al. [12] also found performance improvements in the *n*-back task through WM training. Further studies and reviews that used both *n*-back and other tasks to measure WM performance and near transfer have produced positive results [8,10]. These results can also be complemented by findings in specific population groups such as the elderly [11] or children [35].

Regarding the transfer to episodic memory, only the WM group showed improved performance, while the attention group did not. Thus, it may be assumed that performance increases in the WM group may be attributed to the WM training, and a transfer to episodic memory had taken place. Practice effects, however, still cannot be ruled out due to the absence of a passive control group. These findings coincide with those of other studies that have also used word lists or similar memory tasks. Borella, Cantarella, Caretti, De Lucia, and De Beni [36] found improvements in episodic memory through WM training in the elderly. This is in line with the results of Ball, Berch, Helmers, Jobe, Leveck, Marsiske et al. [37] who also reported transfer to episodic memory in the elderly using a word list task.

Based on our results, an intriguing question is why group-specific effects were observed for the transfer to episodic memory but not for the transfer to the untrained WM task. As stated earlier, the used near transfer WM task clearly demands substantial attentional resources, and both groups improved in performance due to their respective training programs. However, attentional resources are also required to some extent for the untrained episodic memory task, but the attention group did not show performance increases in this task. As mentioned before, it cannot be ruled out that performance increases in the near transfer are due to retest effects. However, because of the group-specific performance benefits in the episodic memory task, mere retest effects appear to be less likely. The episodic memory task of the present study involves a WM component because the words to be remembered occur sequentially. Furthermore, neuroimaging studies suggest that WM and episodic memory tasks activate partly the same brain regions such as the dorsolateral prefrontal cortex and the hippocampus [38]. Interestingly, the PathMemo task of the WM training program was specifically associated with an increase in performance in the episodic memory task. This task combines WM span and visuospatial attention, thus engaging multiple executive and binding processes, which could be beneficial for successfully performing the episodic memory task [39]. Mechanistic explanations of how certain cognitive tasks or task characteristics may affect transfer effects should be investigated in more detail in future studies, e.g., by applying neuroscientific approaches [40].

Regarding the role of the personality traits, that has rarely been addressed in cognitive training to date, a significant positive association of neuroticism was found for the WM training tasks and a statistical trend to a negative relation with the untrained *n*-back transfer task of moderate effect size, whereas no significant association was observed for the untrained word list task. This effect pattern is partly different to a study by Studer-Luethi et al. [24] who showed a significant negative relationship of neuroticism with the overall training mean score in the trained *n*-back tasks (but no significant association with training gain) and a significant negative transfer effect on the improvement in an untrained *n*-back task. The present findings may be consistent with assumptions of attentional control theory (ACT), suggesting that intrusive thoughts and worries in individuals with higher anxiety, a key characteristic of neuroticism, interfere with attentional control resources, thereby potentially limiting WM storage and processing [41]. This has been supported by several behavioral and neuroimaging studies, particularly in terms of demanding tasks [25,42]. Moreover, neurotic individuals are characterized by higher stress reactivity and test anxiety, which may increase with higher task demands and demanding situational factors, thereby negatively affecting their cognitive performance [43,44]. In the present study, the transfer tasks were conducted in a lab situation which can be less controlled by the participants and might thus be experienced as more stressful and worrying for neurotic individuals. According to ACT, this may exert negative effects on WM processing capacity, which may then contribute to the observed negative effect pattern on the *n*-back transfer task. In the domestic setting, in turn, the training situation can be highly controlled by the individuals. This could have a positive effect on performance improvement. Specifically, in stressful situations, anxious individuals try to compensate for potential performance decrements by using strategies or additional effort to achieve task goals [41] but this may fail in the context of multiple stressors in the lab (e.g., performing a demanding cognitive task, performance evaluation by others, presence of experimenters, etc.). This may have contributed to the negative association between neuroticism and the *n*-back transfer task. One might speculate that in a highly controllable domestic setting, but with the presence of a clear task goal, such effortful processing may have positive effects on performance in individuals scoring higher in neuroticism, i.e., contribute to training gains. Although less pronounced, similar effects of neuroticism were also observed in the active control group, including on the *n*-back and word list transfer tasks. Among other potential limitations on the effects of personality outlined below, it is therefore unclear whether the overall results pattern represents true training effects. This needs to be further investigated in future studies. Additionally, in another naturalistic study, no true training effects were found. However, since laboratory studies suggest near transfer effects, it is recommended that more studies should be conducted in this area. This will also provide further meta-analytical findings to consolidate the current state of research [22].

Consistent with the findings of Studer-Luethi et al. [24], conscientiousness was significantly positively associated with performance gains in the *n*-back transfer task, which may indicate that individuals who describe themselves as more conscientious show larger improvement in this transfer task. A positive effect of moderate effect size on performance gains was also observed for the word list transfer task, which, however, was not statistically significant, possibly due to the small sample size, while there were no substantial effects in the active control group. However, for the training tasks, no association with conscientiousness could be observed in the present study, indicating that a potential improvement in the transfer tasks at t1 could not be due to the training. As conscientiousness has been shown to be positively associated with achievement striving, goal orientation, and persistence [45], it seems likely that conscientious individuals were more cognitively motivated to work on the task, potentially contributing to increased improvements. In this context, one may speculate that conscientious individuals show more effort and task adherence in the laboratory situation and in the presence of experimenters than in the domestic setting, which may be one reason for substantial effects in the transfer, but not in the training tasks. Furthermore, it might be that conscientious individuals have better episodic memory, and show stronger practice effects, but there is no association (*p* > 0.80) between conscientiousness and performance on the word list (and *n*-back) task at t0. However, similar to neuroticism, the role of conscientiousness in cognitive training regimes needs further investigation, in particular whether observed effect patterns represent true training effects, that is, plasticity-related changes in the trained function [6] or rather retest/practice or motivation effects [46].

A positive association with task improvement was also expected for individuals with higher NFC scores, who are assumed to be more engaged in effortful cognitive endeavors [47,48]. However, NFC showed no substantial association with improvement in the training and transfer tasks. One may speculate that individuals high in NFC are engaged in complex thinking and ideas instead of being attracted by executive function tasks. In addition, no substantial effects were observed for the other FFM personality traits on WM performance and on improvement in the *n*-back task or the word list task. As outlined above, due to the present sample size, some of the interpreted personality effects did not reach conventional p-thresholds despite moderate effect sizes. As it is recommended to also use effect size estimates for effect interpretation, we interpreted effects of moderate effect size here. Furthermore, we deemed it important to show bivariate and uncorrected associations between personality traits and training/transfer tasks to show the full effects pattern, which may be of interest since there is very little research available on the potential role of personality in cognitive training to date. In this regard, it should be noted that most of the effects in the present study and in relation to personality effects would not withstand a strong conservative Bonferroni correction for multiple comparisons.

The present study had some further limitations. As mentioned above, more valid conclusions would have been possible with the inclusion of a third additional passive control group to identify mere retest effects. The lack of this third group complicates interpretation of the results, especially for the near transfer domain, as outlined above. However, the present study aligns with existing training research in which a large number of studies only used active control groups. In this context, it needs to be mentioned that many previous studies have used no contact control or passive control groups, respectively, instead of active control groups, which is potentially more detrimental to the validity than the lack of an additional passive control group [6,7]. Nonetheless, it is highly recommended for future studies to implement active and passive control groups. A further limitation concerns the sample. Since the participants were all psychology students, the generalizability of the study must be critically questioned. Upcoming studies should therefore try to collect more representative samples.

The naturalistic setting of the study may have led to a further limitation. Compared to lab-based training, the domestic training setting may have led to a higher variance in training participation, reflecting a natural variance. To create as much similarity as possible to everyday training situations, predefined programs of commercial brain training, as available to any user, were selected. However, this means that it is difficult to separate the individual cognitive functions by task type. Individual working memory tasks can also be found in the attention program and vice versa, in fact, three tasks have been part of both training programs. This can hardly be disentangled but could offer one explanation why both groups benefit in the area of near transfer.

In conclusion, true training effects associated with improvement in episodic memory may have practical implications, from small everyday improvements to interpersonal changes. However, these results are not inconclusive and are controversial in the literature. Further studies in this area should focus on identifying possible true training effects and plasticity-related changes in order to identify the relevance of specific brain training types and possible transfer effects. The potential effects of personality traits on training and transfer tasks suggest that training and transfer effects may vary with the level of these traits. Future research, however, should further examine whether the observed effects reflect true training and transfer effects.

## Figures and Tables

**Table 1:** Descriptive statistics. *M* = Mean, *SD* = Standard Deviation, f = Female, m = Male, *n*-back Acc = Accuracy in *n*-back task, Word list t0 = Correctly remembered words at baseline.

	Experimental Group	Control Group	Mean Absolute Difference
Age	*M* = 21.83 (*SD* = 1.69)	*M* = 22.12 (*SD* = 1.74)	*t*(52) = −0.63, *p* = 0.84
Gender	24 f/5 m	18 f/7 m	*c*²(1, *n* = 54) = 0.90, *p* = 0.51
Training Units	*M* = 17.34 (*SD* = 6.58)	*M* = 17.28 (*SD* = 5.99)	*t*(52) = 0.038, *p* = 0.50
*n*-back Acc t0	*M* = 16.55 (*SD* = 20.80)	*M* = 22.12 (*SD* = 21.45)	*t*(52) = −0.97, *p* = 0.34
Word list t0	*M* = 7.60 (*SD* = 1.75)	*M* = 6.77 (*SD* = 1.62)	*t*(52) = 1.8, *p* = 0.78

## Data Availability

The data presented in this study are available on request by the corresponding author.

## Supplementary Materials

The following are available online at https://www.mdpi.com/article/10.3390/brainsci11081083/s1, Table S1: NeuroNation training tasks, Figure S1: Improvement in NeuroNation tasks.
