# Supplementary material for: Commercial Brain Training: Efficacy, Transfer Effects, and the Influence of Personality Traits: A Study Conducted on Healthy Young Adults
Source: Brain Sci. 2021 Aug 18;11(8):1083. doi: 10.3390/brainsci11081083 (PMC8392474; doi:10.3390/brainsci11081083)

Supplementary Materials: brainsci-1274903

Supplementary Table S1: NeuroNation training tasks

Task	Description	Training Program	Performed at least 6 times by n participants	Graphics
BinMemo	Here the balls in each box must be counted and the information about how many balls are in each box must be constantly updated.	WM program	26	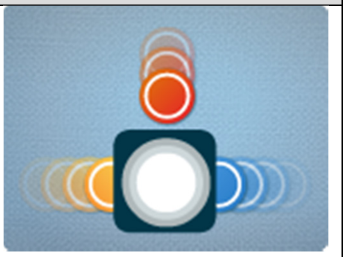
PathMemo	Moving circles must be tapped in the displayed order.	WM program	25	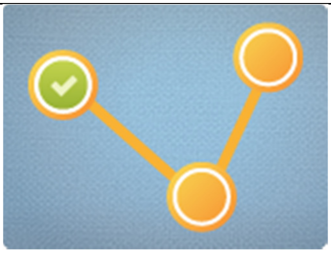
Shuffler	It must be remembered on which places the individual figures stand, whereby they can change their places concealed.	WM program	24	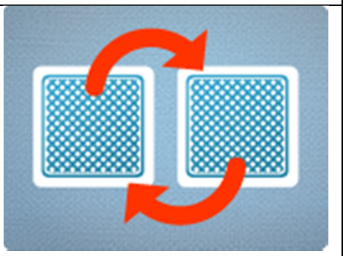
Symbolski	In the Pattern Finder exercise, the challenge is to perform a memory task (assigning a number to a symbol). By changing the places of the symbols and letters, it is more difficult to memorize the number-symbol-combination. Since the memorization does not take place in the central field of vision, the exercise also trains the processing speed.	WM program	21	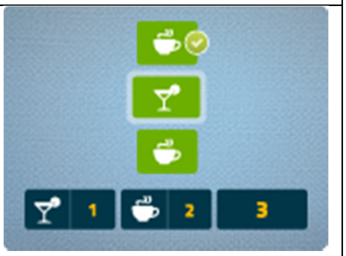

FormFever	One must decide whether the displayed pattern corresponds to the displayed description and the impulse to answer quickly must be suppressed in favor of a correct answer.	WM program	28	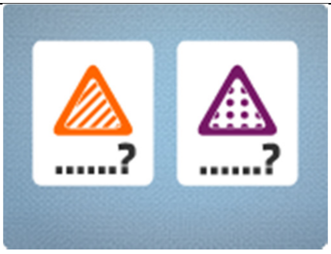
DynamicSequence	The following of a displayed number must be found.	Attention program	22	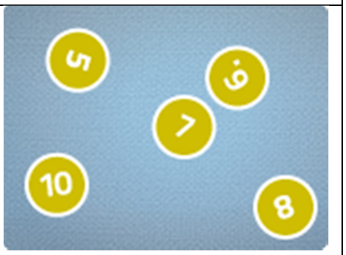
IntruderCraft	The task is to detect minimal differences in a large number of strongly similar shapes.	Attention program	22	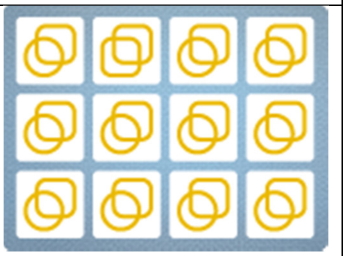
Mackworth	The questions asked in the middle of a clock must be answered by following the movement of the clock hand.	Attention program	25	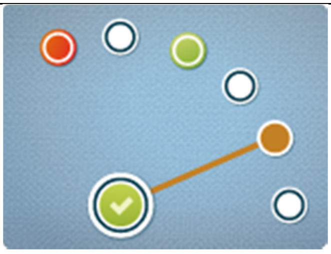
MathChain	Through mathematical exercises, basic arithmetic and memory are trained.	Attention program	22	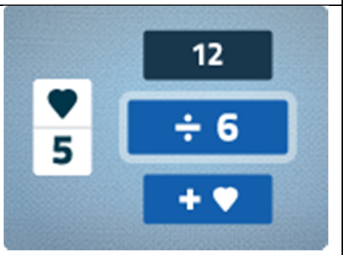
ParaChain	In each box, a mental calculation task must be executed in parallel, i.e independently of the other boxes, while constantly keeping the last results of each box in memory.	Attention program	20	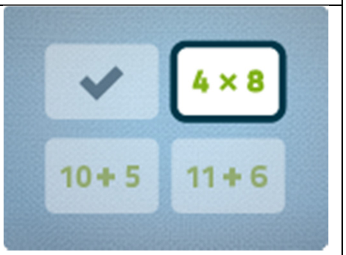

ColorCraze	Symbols and colors must be named correctly, while content and color are displayed differently (Stroop test).	WM and attention program	48	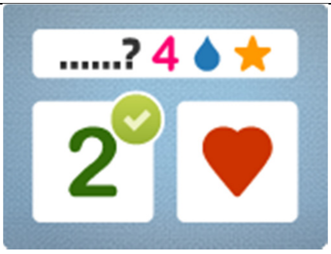
PathPredictor	The challenge in searching for clues is to implement the route description in the mind's eye. At the same time, the route already taken must be temporarily stored in the working memory.	WM and attention program	39	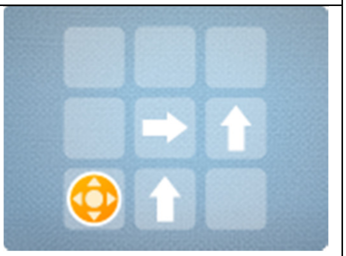
Rotator	You must select the pattern that matches the one shown in the center.	WM and attention program	46	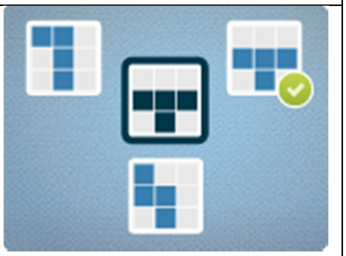

<u>Supplementary figure S2:</u> Improvement in NeuroNation tasks

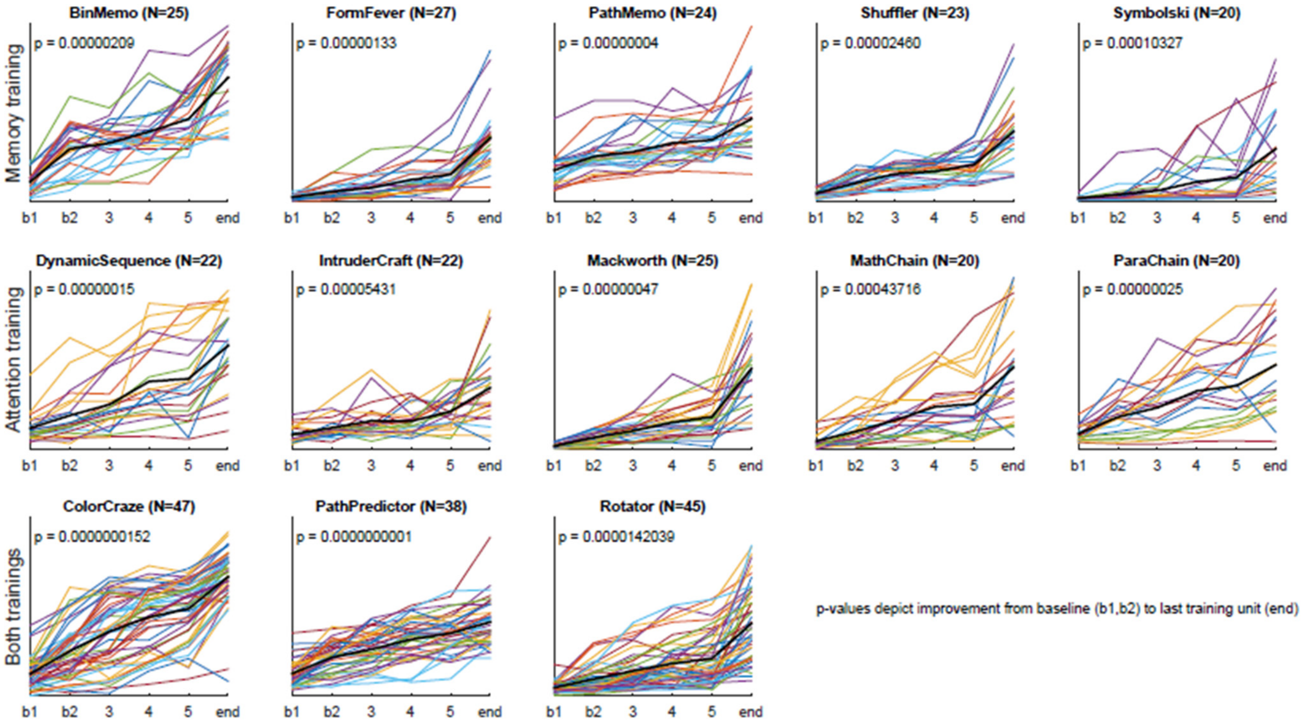

Supplement: Supplementary file 1 [file brainsci-11-01083-s001.zip › brainsci-1274903-supplementary.pdf]
